# Gene Identification and Characterization of Correlations for DEPs_DEGs Same Trend Responding to Salinity Adaptation in *Scylla paramamosain*

**DOI:** 10.1155/2019/7940405

**Published:** 2019-02-10

**Authors:** Huan Wang, Hongling Wei, Lei Tang, Junkai Lu, Changkao Mu, Chunlin Wang

**Affiliations:** ^1^School of Marine Science, Ningbo University, Ningbo, 315211 Zhejiang, China; ^2^Key Laboratory of Applied Marine Biotechnology, Ministry of Education, Ningbo University, Ningbo, 315211 Zhejiang, China

## Abstract

*Scylla paramamosain* is a commercially important species distributed along the coast of southern China and other Indo-Pacific countries. Sudden salinity drop exceeding the adjustment capability of *S. paramamosain* can result in damage or even mortality. In our previous study, we had analyzed the mechanism of adapting sudden drop in salinity from the level of transcriptomics and proteomics, respectively. This study performed a correlation analysis of RNA sequencing transcriptomics and iTRAQ proteomics in order to investigate the adaptation mechanisms to sudden salinity drop from 23‰ to 3‰. There were 3954 correlations and a total of 15 correlations for differentially expressed proteins (DEPs) and differentially expressed genes (DEGs) from proteomics and transcriptomics. The correlation between DEPs and DEGs was 0, and the Spearman correlation coefficient of the same trend correlation for DEPs and DEGs was the highest, reaching 0.9080. KEGG pathway enrichment correlation revealed that protein digestion and absorption (Ko04974), proximal tubule bicarbonate (Ko04964), and bile secretion (Ko04976) played important roles in Na^+^/H^+^ and Na^+^/K^+^ exchange. In addition, important genes related to osmoregulation, such as ion transport and carbonic anhydrase, were also detected in the correlation analysis for same trend DEPs_DEGs. In conclusion, the proteome and transcriptome correlation results from this study indicate that ion transport plays a critical role in the adaptation of *S. paramamosain* to sudden reduction in salinity.

## 1. Background

*Scylla paramamosain* is a large marine crustacean widely distributed along the coast of southern China and other Indo-Pacific countries [1, 2]. It is the dominant species of *Scylla* on the southeast coast of China [3]. It is a very important economic species along the southeast coast of China due to its large size, fast growth, palatability, and nutritional value. *Scylla paramamosain* is a euryhaline species, especially in shallow sea and nearshore estuary habitats. Previous studies have shown that the salinity tolerance ranges from 40‰ to 0 [4–8]. Some productive experiments have been carried out and indicated that *Scylla paramamosain* can survive and grow normally through the salinity gradually decreases.

Osmoregulation is an important function that allows marine animals to adapt to salinity changes in seawater environments [9–11]. Salinity is a key abiotic parameter that influences the distribution, abundance, physiology, and health of crustaceans [12–15]. Although *S. paramamosain* is a euryhaline species [16, 17], significant changes occur in immune-related enzyme activity under the stress of salinity, prolonged stress time, serum PO level, and SOD in muscles and the hepatopancreas, which affect its immunity [18]. Furthermore, changes in salinity can lead to increased O_2_ consumption in shrimps and crabs, increased energy requirements, and accelerated metabolism, consequently resulting in physiological dysfunction and reduced immune defense capacity [19, 20]. In this case, latent pathogenic bacteria in the body or environment may invade and result in mortality. Although *S. paramamosain* has strong osmoregulatory capacity and can almost be cultured in freshwater aquaculture in some areas of China (such as Shanghai and Guangzhou), it is still extremely sensitive to sudden reductions in salinity, especially sharp falls (10‰ based on production data). One example of a scenario causing a sudden reduction in salinity is heavy rainfall in ponds, which leads to *S. paramamosain* mortality. In recent years, several studies have been published on the salinity tolerance of *S. paramamosain* ([6, 21]; Hai et al., 1998; [22–24].).

Transcriptomics focuses on gene transcription and regulation of transcription in cells [25, 26]. The main tool used in transcriptomics is total RNA sequencing, which is a powerful tool for analyzing gene expression changes in response to various environmental stresses [27]. Proteomics is a new science direction that explores biological activity related to protein expression [28]. It is a comprehensive science focusing on translational changes, posttranslational modifications, and interactions among protein molecules. Isobaric tags for relative and absolute quantification (iTRAQ) is a new protein quantification technology based on isotope labeling combined with multidimensional liquid chromatography and tandem mass spectrometry (MS) [29, 30].

The central dogma explains the information flow of gene expression: “gene>mRNA>protein.” Genes are subject to multiple levels of regulation in this process. To date, the consistency of expression between mRNA and corresponding proteins has been reported to be low. Therefore, proteomics and transcriptomes are jointly analyzed to help identify gene expression regulation [31]. At present, the *S. paramamosain* genome has not yet been completed, and the combined analysis of histology and multiomics is a suitable approach for studying differences in target features. In previous studies, separate analyses were performed at transcriptome and protein levels. The analysis of transcriptome implied that ion transport and amino acid metabolism were key factors in regulating the salinity adaptation of *S. paramamosain*, but ion transport might be more important than amino acid metabolism in the regulatory process [32]. However, the results of proteome implied that amino acid metabolism played a more important role in the process of adaption for *Scylla paramamosain* [33]. So, in this study, a correlation analysis of RNA sequencing transcriptomics and iTRAQ-tandem MS proteomics was used to further explore the key genes of correlations for DEPs_DEGs Same Trend and their involved molecular mechanisms responding to salinity adaptation in *Scylla paramamosain*.

## 2. Methods

### 2.1. Treatment

A total of 300 randomly selected crabs (from our artificial breeding) with a body weight of ~30 g were selected and kept in a natural water environment with a salinity of 23‰ and a temperature of approximately 20°C. Every 50 crabs were randomly selected (weight ~30 g) as a group, with a total of six groups, housed in six cement pools under identical physical and chemical conditions. The seawater salinity for three of the groups was adjusted to 3‰ from 23‰, a reduction of 20‰. These three groups were defined as the low-salinity (LS) group. The other three groups were defined as the CK groups, in which the salinity of seawater was maintained at 23‰. All other parameters in this group were the same as in the LS group [32, 33].

### 2.2. Total RNA Extraction and Transcriptome Sequencing

Total RNA was extracted from the gill tissue of *S. paramamosain* using RNAiso Plus (TaKaRa, Dalian, China) according to the manufacturer's instructions. For Illumina paired-end sequencing, equivalent quantities of total RNA isolated from the three mud crabs were pooled as one sample, resulting in three samples per group (CK and LS). The transcriptome sequencing was performed by BGI (Shenzhen, China) [32].

### 2.3. Total Protein Extraction and Proteome Sequencing

The protein extraction is referred in [33]. For iTRAQ assays, equivalent quantities of total protein isolated from the three mud crabs were pooled as one sample, resulting in three samples per group (CK and LS). The proteome sequencing was performed by BGI (Shenzhen, China) [33].

### 2.4. Correlation Analysis of Proteome and Transcriptome Parameters

In the correlation analysis, the transcriptome and proteome data required processing and integrating in various ways. Correlation analysis data screening and difference definitions are shown in Table 1.

## 3. Results

### 3.1. Correlation Identification and Characterization

The seawater salinity for *S. paramamosain* in the CK group was 23‰. A total of 150 individuals were randomly selected from the CK group and placed in a 3‰ salinity seawater environment as the LS group. A previous study showed that after 120 h, *S. paramamosain* in the LS group started to adapt to a low-salt (3‰) seawater environment with normal diet and activities. At 120 h [32], *S. paramamosain* from the CK and LS groups were taken for transcriptomics and proteomic analysis.

**Figure 1:**
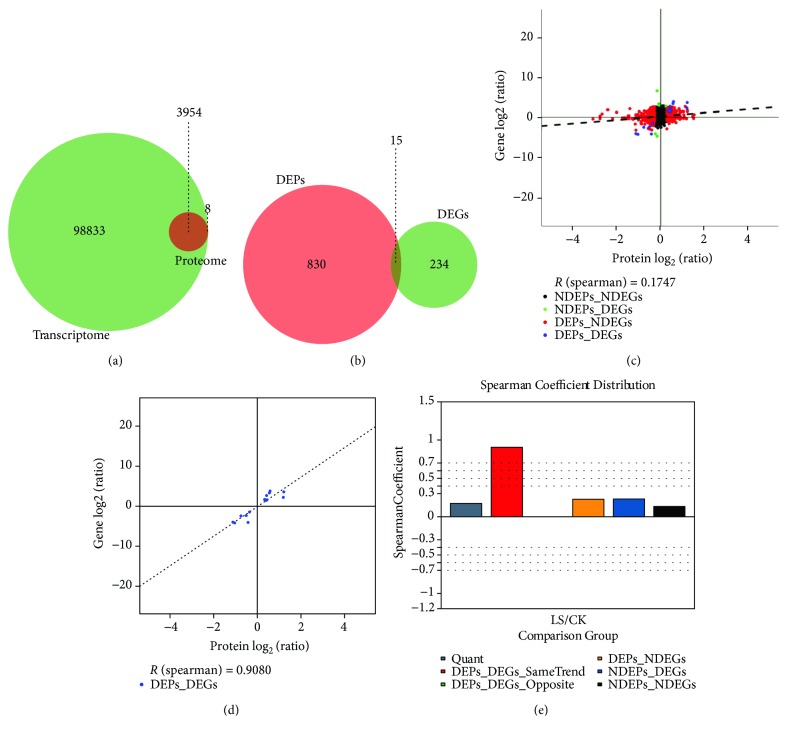
Correlation statistics. (a) Venn diagram showing number of correlations at identification level for proteome and transcriptome. Green circles indicate transcriptome; red circles indicate proteome; (b) Venn diagram showing number of correlations at identification level for DEPs and DEGs; (c) correlation analysis at quantitative level. Red dots indicate DEGs; green dots indicate DEPs; (d) differential correlations analysis; (e) correlation coefficients of all correlation analysis results. *R* (Spearman)_Quant = 0.1747, *R* (Spearman)_DEPs_DEGs_Same Trend = 0.9080, *R* (Spearman)_DEPs_DEGs_Opposite = 0, *R* (Spearman)_DEPs_NDEGs = 0.2292, *R* (Spearman)_NDEPs_DEGs = 0.2319, *R* (Spearman)_NDEPs_NDEGs = 0.1441. NDEPs and NDEGs represent no DEPs and no DEGs, respectively.

Transcriptome and proteome analyses detect mRNA and protein expression levels in specific organisms, tissues, cells, or organelles. In the correlation analysis, when the expression of protein is detected at the transcriptome level, it is considered correlated. At the transcriptional level, 102,787 genes were identified and quantified (Figures 1(a) and 1(c)); at the protein level, 3962 proteins were identified and quantified (Figures 1(a) and 1(c)); a total of 3954 correlations were identified and only eight protein molecules were found to be uncorrelated (Figures 1(a) and 1(c)). At the transcription level, 249 CK vs. LS differentially expressed genes (DEGs) were obtained (fold change ≥ 2.00 and *P* value ≤ 0.05) (Figure 1(b)), whereas 845 CK vs. LS differentially expressed proteins (DEPs) were screened at the protein level (fold change ≥ 1.2 and *P* ≤ 0.05) (Figure 1(b)). Moreover, there were 15 correlations for DEPs and DEGs from proteomics and transcriptomics analyses (Figures 1(b) and 1(d)). Correlation analysis showed that there was no opposite correlation for DEPs and DEGs (Figure 1(e)), and Spearman correlation coefficient of the same trend correlation for DEPs and DEGs was the highest, reaching 0.9080 (Figure 1(e)).

**Figure 2:**
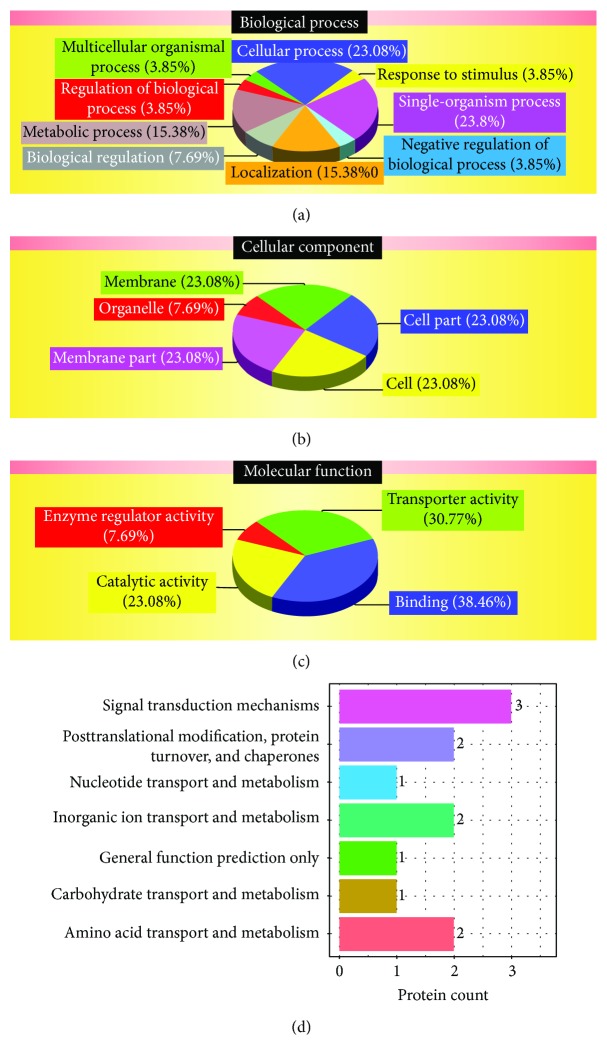
GO analysis and COG function classification of correlations for DEPs_DEGs_Same Trend. (a) Biological process; (b) cellular component; (c) molecular function; (d) COG function annotation and classification. GO analysis was performed with the correlative proteins using Blast2GO [49]. COGs were delineated by comparing protein sequences encoded in complete genomes, representing major phylogenetic lineages. Each cog consists of individual proteins or groups of paralogs from at least three lineages and thereby corresponds to an ancient conserved domain. Each cog consists of individual orthologous proteins or orthologous sets of paralogs from at least three lineages. Orthologs typically have the same function, allowing transfer of functional information from one member to an entire cog. This relation automatically yields a number of functional predictions for poorly characterized genomes. The COGs comprise a framework for functional and evolutionary genome analysis [50, 51].

Gene ontology (GO) analyses of correlations for DEPs_DEGs_Same Trend showed that the proteins could be categorized into several biological processes, i.e., cellular process (23.08%), single-organism process (23.08%), metabolic process (15.38%), localization (15.38%), and biological regulation (7.69%) (Figure 2(a)). The cellular components identified by protein GO analysis are depicted in Figure 2(b). The major molecular functions identified by protein GO analysis were binding (38.46%), transporter activity (30.77%), catalytic activity (23.08%), and enzyme regulator activity (7.69%) (Figure 2(c)). GO analyses of correlations for Quant, DEPs_NDEGs, and NDEPs_NDEGs are shown in Figure S1–S3, respectively. However, correlations for NDEPs_DEGs contained many proteins, which had no annotation in the GO analysis. Cluster of Orthologous Groups of proteins (COG) function classification of correlations for DEPs_DEGs_Same Trend indicated that the proteins can be classified into several functions, i.e., signal transduction mechanisms (3 proteins), posttranslational modification, protein turnover, chaperones (2 proteins), inorganic ion transport and metabolism (2 proteins), amino acid transport and metabolism (2 proteins), nucleotide transport and metabolism (1 protein), general function prediction only (1 protein), and carbohydrate transport and metabolism (1 protein) (Figure 2(d)). Correlations between the other groups are shown in Figure S4.

### 3.2. Proteome and Transcriptome in Metabolic Pathway Correlation Analysis

**Figure 3:**
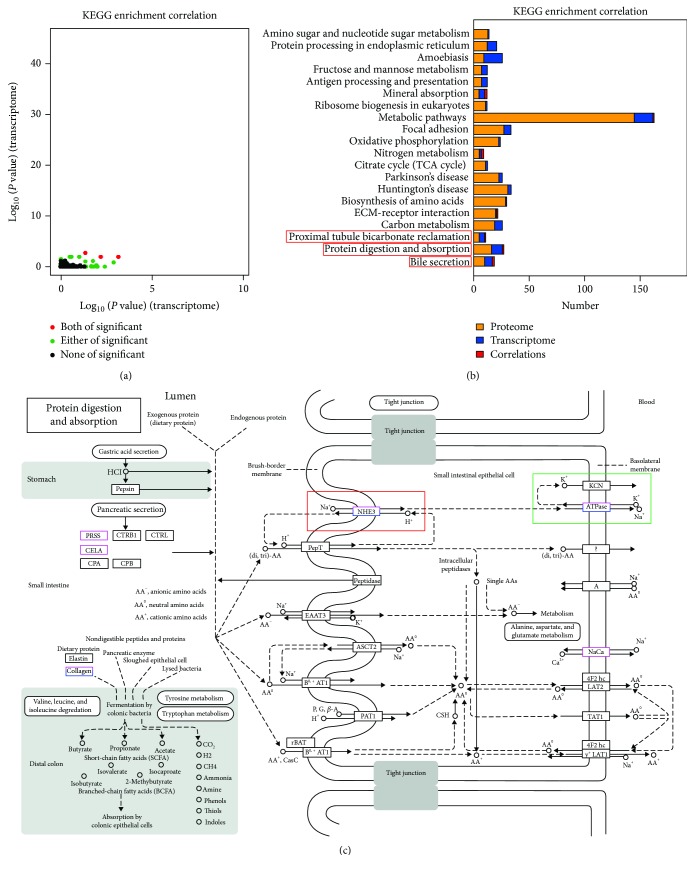
KEGG pathway enrichment correlation. (a) Pathway enrichment correlation analysis. (b) Pathway correlation statistics of the top 20 pathways from proteome. (c) Protein digestion and absorption (Ko04974). The red box represents Na^+^/H^+^ exchange, and the green box represents Na^+^/K^+^ exchange. Significance: both proteome and transcriptome groups have significant enrichment in pathways listed in the table through the hypergeometric test. The significance threshold is *P* value < 0.05.

Simultaneous annotation of transcriptome and proteome data in metabolic pathways can facilitate the formation of the overall view of gene expression. The correlation analysis revealed 152 correlations (Table S1) and three significant enrichment correlations (Figure 3(a) and Table S1): protein digestion and absorption (Ko04974), proximal tubule bicarbonate (Ko04964), and bile secretion (Ko04976) (Figure 3(b) and Table 2). To date, there has been no report on a direct correlation between these three metabolic pathways and osmoregulation. However, the results of the present study showed that the three pathways were significantly enriched at proteome and transcriptome levels, suggesting that these three pathways may have a function in *S. paramamosain* osmoregulation and adaptation to low salinity. The low salinity adaptation process of *S. paramamosain* is an important manifestation of its osmoregulation function. Analysis of the metabolic network of pathway protein digestion and absorption (Ko04974) showed that this pathway was closely related to Na^+^ exchange (Figure 3(c)), while Na^+^ performs important functions in osmoregulation of crustaceans. Crustaceans utilize Na^+^/K^+^-ATPase, V-ATPase, and other ion channels located in the plasma membrane of enamel epithelium to regulate hemolymph osmotic pressure with the surroundings in order to achieve salinity adaptation through Na^+^/K^+^ exchange, Na^+^/H^+^ exchange, and Cl^−^/HCO_3_^−^ exchange. In pathways that were significantly enriched in this study, protein digestion and absorption (Ko04974) and proximal tubule bicarbonate (Ko04964) were closely related to Na^+^/H^+^ and Na^+^/K^+^ exchange (Figure 3(c) and Figures S5 and S6). Therefore, the above pathway enrichment analysis suggests that changes in surrounding salinity alters osmotic pressure, and *S. paramamosain* epithelial cells achieve hemodialysis in vivo through Na^+^/H^+^ and Na^+^/K^+^ exchange.

### 3.3. Analysis of Correlations for DEPs_DEGs Same Trend

The regulation of eukaryotic gene expression comprises multiple complex levels including transcription, posttranscriptional, and translation levels. Therefore, the regulation of gene/protein from correlations of DEPs_NDEGs, NDEPs_DEGs, and NDEPs_NDEGs requires a more in-depth exploration. In the following context, this study focused on analysis of gene/proteins from correlations for DEPs_DEGs (because correlations for DEPs_DEGs opposite is 0; DEPs_DEGs refers to the DEPs_DEGs Same Trend).

**Figure 4:**
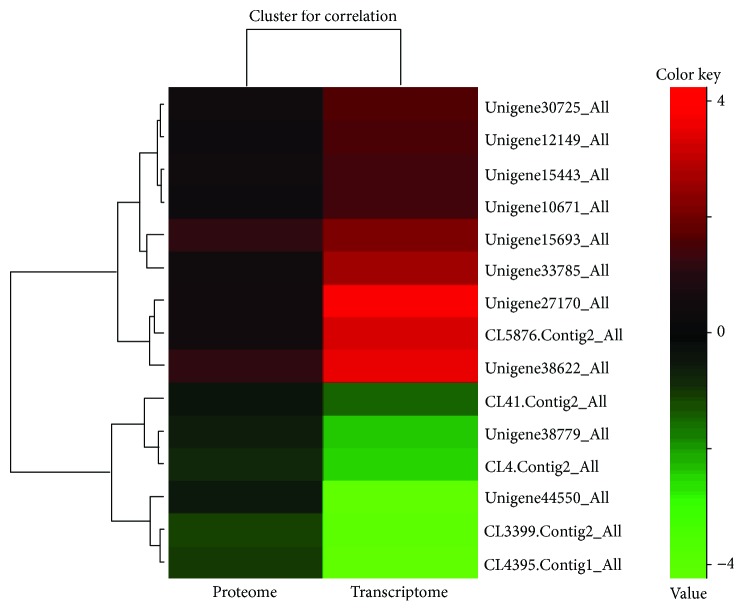
DEPs and DEGs cluster analysis. Each row in the diagram represents a protein/gene, with different colors representing different multiples. Red and green represent upregulated and downregulated expression, respectively.

A cluster analysis for DEPs and DEGs revealed eight upregulated genes/proteins and six downregulated genes (Figure 4 and Table 3). Annotation showed that upregulated genes/proteins were mainly related to ion channels; for example, Unigene38622_All is annotated as “sodium/hydrogen exchanger,” CL5876.Contig2_All is annotated as “sodium- and chloride-dependent glycine transporter,” and Unigene15443_All is annotated as “chloride channel” (Table 3). This result is consistent with classical studies of osmoregulation in crustacean gills by ion transport [34, 35]. Of the downregulated genes/proteins, CL3399.Contig2_All is annotated as “glutamine synthetase,” CL4395.Contig1_All is “mannose-binding protein”, Unigene5126_All is “protein takeout”, and CL41.Contig2_All is “urea transporter” (Table 3). KEGG pathway classification and annotation of correlations for DEPs and DEGs Same Trend showed that, in addition to ion transport and other related metabolism, amino acid metabolism may also be involved in the regulation of low-salt adaptation in *S. paramamosain* (Table 4) [36, 37].

### 3.4. Validation of Gene Expression by qPCR

**Figure 5:**
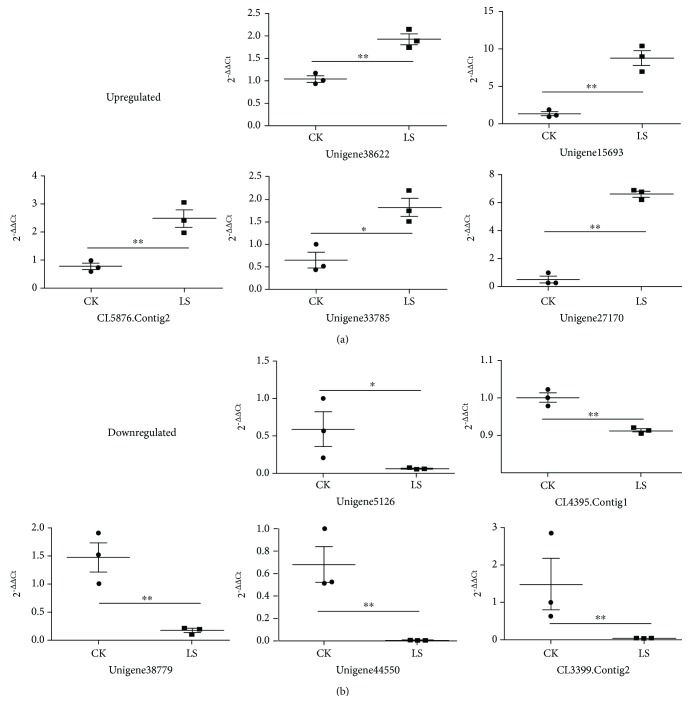
Validation of gene expression by qPCR. ^∗^ *P* < 0.05 and ^∗∗^ *P* < 0.01.

Ten arbitrarily selected DEGs from correlations for DEPs_DEGs Same Trend encoding sodium/hydrogen exchanger (Unigene38622_All), carbonic anhydrase (Unigene15693_All), no description (Unigene27170_All), sodium- and chloride-dependent glycine transporter (CL5876.Contig2_All), no description (Unigene33785_All), no description (Unigene44550_All), no description (Unigene38779_All), protein takeout (Unigene5126_All), mannose-binding protein (CL4395.Contig1_All), and glutamine synthetase (CL3399.Contig2_All) were selected for qPCR to validate the DEGs (Figure 5). The trend of gene expression was exactly the same between qPCR and RNA-seq results (Figure 5 and Table 3).

## 4. Discussion

Traditional molecular biology suggests that one gene encodes one protein molecule, one protein has one structure, and one structure performs one function, following the dogma that information flows from sequence to structure and further to function. Function performed in vivo is often in a regulation network of biological systems. Exploration of a single gene and/or protein is conducive to in-depth study of gene functions and products in cells. However, these studies contain many deficiencies: interactions with other genes and proteins are neglected, reproducibility of gene and protein detection in different physiological conditions or cells is unacceptable, and the functions of a single gene or protein cannot explain a certain biological characteristic. An increasing number of studies have shown that gene expression alone rarely dominates in the occurrence of a biological event. The occurrence of an event is the simultaneous expression of a group of genes and the synergy of a group of proteins. Therefore, in fact, a more accurate representation of biological functions should comprise a set of related genes and a set of interacting proteins, which form an interactive network. In this case, a more accurate representation of biological functions should comprise a network of interacting biological macromolecules. This is a new research direction in system biology [38]. Gene set enrichment analysis is a defined gene set-based method that overcomes many deficiencies of single-gene analysis [39]. To date, commonly used gene set databases comprise GO and KEGG. For multiomics data, GO and KEGG pathways are enriched at transcriptome and proteome levels, and the two sets of data are combined for further analysis, facilitating the study of gene expression regulation from the perspective of gene set coexpression [40]. The present study was based on a correlation analysis of transcriptomic and proteomic genomics data. Due to the complexity, it is challenging to analyze genes with different expression trends in one histological analysis. Therefore, this study focused on single gene/protein and gene set enrichment analyses in order to detect correlations between DEPs and DEGs.

Researches show that a preliminary experiment was performed to select a salinity for treatment, including 13‰, 8‰, 5‰, 3‰, and 1‰, and the degree of salinity drop was 10‰, 15‰, 18‰, 20‰, and 22‰, respectively. Finally, crabs in salinity 3‰ begin to die, and most crabs nearly died in salinity 1‰. So 3‰ salinity might be the optimal choice as a critical point for the research of adaptive mechanism responding to sudden drop in salinity [32]. In this study, there were 4 dead crabs in the CK group and 24 dead crabs in the LS group, in which each group had 150 crabs before the beginning of the experiment. The environment under the experiment condition is well controlled, so the number of deaths is not too great. However, the pond environment is extremely complicated under natural conditions and would lead to more deaths and production loss.

Salinity is an important factor impacting the growth, development, and health of aquatic crustaceans. Previous studies have shown that low salinity can influence ion channels [15, 34, 35] and free amino acids [36, 37], which are related to osmoregulation. The ion transporter-type gill epithelium of crustacean is the main site for osmotic regulation and ion transport, and the regulation of ion transport is mainly accomplished by the action of many ion transporter enzymes, such as Na^+^-k^+^-ATPase, V-ATPase, HCO_3_^−^-ATPase, and CA (carbonic anhydrase) [41]. Dalla Via [42] found that the content of free amino acids in the hemolymph of *Palaemon elegans* increased by nearly 55% after entering the high-salinity environment from low salinity. In the freshwater and low-salinity environment, the total free amino acids of the hemolymph in *Macrobrachium rosenbergii* are only 0.85~1.0 mmol·L^−1^, while in high salinity, the total free amino acid rose sharply to 2.1 mmol·L^−1^, where alanine is 6 times higher than in freshwater and low-salt environments [43]. Dalla Via [44] found that the free amino acids in *Litopenaeus vannamei* and *Penaeus japonicus* vary linearly along with the change in salinity; the main free amino acids are glycine, taurine, arginine, proline, and alanine, but the main osmotic pressure effector is glycine, proline, and alanine. This shows that free amino acids are important effects in the osmotic pressure of crustaceans. In the present study, a series of upregulated ion channel-related genes were discovered via genes/proteins from correlations for DEPs and DEGs Same Trend, including Unigene38622_All (sodium/hydrogen exchanger), CL5876.Contig2_All (sodium- and chloride-dependent glycine transporter), and Unigene 15443_All (chloride channel). These genes have shown important salinity regulation functions in classical studies of osmoregulation in aquatic animals [34, 35]. Furthermore, an upregulated expression of Unigene15693_All (carbonic anhydrase), which plays an important role in the osmotic regulation of crustaceans [45–47], was also found in the present study. Moreover, some proteases or inhibitors play important functions, i.e., Unigene10671_All, which is annotated as “serine proteinase inhibitor” and has been reported to be involved in the natural immune response of *S. paramamosain* [48]. The present results suggest that the immune function of *S. paramamosain* is affected following a sudden reduction in salinity from 23‰ to 3‰, consistent with previously reported results. In addition, Unigene12149_All, annotated as “cystatin A precursor,” and three genes: Unigene27170_All, Unigene30725_All, and Unigene33785_All, are unknown genes. Interestingly, of the upregulated genes/proteins, the gene ratio (LS/CK)/protein ratio (LS/CK) ranged from 2.03 to 9.34, while the downregulated ratio ranged from 0.08 to 0.47. This result indicates that in the upregulated expression, transcription factors are upregulated by a factor that is greater than that of protein upregulation, while in the downregulated expression, transcription is downregulated by a factor that is less than that of protein downregulation.

Moreover, KEGG pathway analysis of correlations for DEPs and DEGs Same Trend integrates single gene/protein analysis at the gene set level and reveals several metabolic pathways that are closely related to osmoregulation. Three pathways, protein digestion and absorption (Ko04974), proximal tubule bicarbonate (Ko04964), and bile secretion (Ko04976), were obtained from the KEGG pathway enrichment correlation of proteome and transcriptome. These are closely related to Na^+^/H^+^ and Na^+^/K^+^ exchange in the plasma membrane of crustacean gill epithelial cells and are one of the most important mechanisms of osmoregulation. In addition, free amino acids [36, 37] are another significant pathway for crustacean osmoregulation; however, pathways for free amino acids [36, 37] from the KEGG pathway enrichment correlation were not found. However, some of the pathways related to amino acid metabolism were discovered in a separate analysis of proteome and transcriptome. Therefore, there are some deficiencies in the multiomics correlation analysis, which requires individual histology analysis combined with multiomics correlation analysis.

In our previous study, we had analyzed the mechanism of adapting sudden drop in salinity from the level of transcriptomics and proteomics, respectively. However, we discovered another new finding through the correlation analysis of proteome and transcriptome. In conclusion, this study identified several key metabolic pathways involved in the adaption of *S. paramamosain* to sudden reductions in salinity, through correlation analysis of proteome and transcriptome. Protein digestion and absorption (Ko04974), proximal tubule bicarbonate (Ko04964), and bile secretion (Ko04976) were found through the KEGG pathway enrichment correlation, which play an important function in Na^+^/H^+^ and Na^+^/K^+^ exchange. In addition, important genes related to osmoregulation, such as ion transport and carbonic anhydrase, were detected in the analysis of correlations for DEPs_DEGs Same Trend. In summary, proteome and transcriptome correlation results indicate that ion transport plays an extremely important role in the adaptation of *S. paramamosain* to sudden reductions in salinity. These findings different from the standalone analysis from level of transcriptomics and proteomics, respectively, provide novel insight into the adaptation of *S. paramamosain* to sudden reductions in salinity.

## Figures and Tables

**Table 1:** Correlation analysis data screening and difference definitions.

Type	Value	Type	Value
Protein_Unique peptide	1	GO_Significant	<0.05
Protein_FoldChange	1	Pathway_Significant	<0.05
Gene_Significant	<0.05	Blast_Identity	100
Gene_FoldChange	2	Blast_Evalue	1e-8

**Table 2:** Pathway enrichment correlation analysis (LS-VS-CK).

ID	Pathway name	Number of proteins	Number of genes	Number of correlations	Significance	
					*P* value (proteome)	*P* value (transcriptome)
Ko04974	Protein digestion and absorption	16 (IDs)	10 (IDs)	1 (IDs)	0.0065	0.0126
Ko04964	Proximal tubule bicarbonate reclamation	5 (IDs)	5 (IDs)	1 (IDs)	0.0456	0.0020
Ko04976	Bile secretion	10 (IDs)	7 (IDs)	2 (IDs)	0.0007	0.0126

Significance indicates that both proteome and transcriptome are significantly enriched; the significance threshold is *P* value < 0.05.

**Table 3:** Description of DEPs and DEGs Same Trend correlations.

Correlation ID	Unique Pep number	Protein ratio (LS/CK)	Gene ratio log_2_ (LS/CK)	DEPs/DEGs	Functional description
Unigene38622_All	14	2.3	3.57	Up	Sodium/hydrogen exchanger
Unigene15693_All	7	2.28	2.21	Up	Carbonic anhydrase
Unigene27170_All	3	1.49	3.80	Up	NA
CL5876.Contig2_All	3	1.47	3.31	Up	Sodium- and chloride-dependent glycine transporter
Unigene15443_All	8	1.36	1.49	Up	Chloride channel
Unigene30725_All	9	1.36	1.71	Up	NA
Unigene33785_All	1	1.34	2.62	Up	NA
Unigene10671_All	11	1.3	1.40	Up	Serine proteinase inhibitor
Unigene12149_All	6	1.26	1.61	Up	Cystatin A precursor
CL41.Contig2_All	2	0.78	−1.46	Down	Urea transporter
Unigene44550_All	3	0.75	−4.08	Down	NA
Unigene38779_All	6	0.7	−2.37	Down	NA
Unigene5126_All	5	0.59	−2.40	Down	Protein takeout
CL4395.Contig1_All	6	0.49	−4.17	Down	Mannose-binding protein
CL3399.Contig2_All	2	0.46	−4.01	Down	Glutamine synthetase

DEPs: fold change ≥ 1.2 and *P* ≤ 0.05; DEGs: fold change ≥ 2.00 and *P* value ≥ 0.05 or fold change ≤ 0.50 and *P* value ≤ 0.05. “NA” shows no description. Gene ratio (LS/CK) = log_2_ fold change (LS/CK).

**Table 4:** KEGG pathway classification and annotation of DEPs and DEGs Same Trend correlations.

NO	Pathway	DEPs with pathway annotation (14)	ID	NO	Pathway	DEPs with pathway annotation (14)	ID
1	Ribosome biogenesis in eukaryotes	2 (14.29%)	Ko03008	16	Renal cell carcinoma	1 (7.14%)	Ko05211
2	HTLV-I infection	2 (14.29%)	Ko05166	17	Amoebiasis	1 (7.14%)	Ko05146
3	Mineral absorption	2 (14.29%)	Ko04978	18	Biosynthesis of amino acids	1 (7.14%)	Ko01230
4	Bile secretion	2 (14.29%)	Ko04976	19	Wnt signaling pathway	1 (7.14%)	Ko04310
5	Nitrogen metabolism	2 (14.29%)	Ko00910	20	Thyroid hormone signaling pathway	1 (7.14%)	Ko04919
6	Purine metabolism	1 (7.14%)	Ko00230	21	HIF-1 signaling pathway	1 (7.14%)	Ko04066
7	Insulin secretion	1 (7.14%)	Ko04911	22	Metabolic pathways	1 (7.14%)	Ko01100
8	Protein digestion and absorption	1 (7.14%)	Ko04974	23	Glyoxylate and dicarboxylate metabolism	1 (7.14%)	Ko00630
9	Central carbon metabolism in cancer	1 (7.14%)	Ko05230	24	Adipocytokine signaling pathway	1 (7.14%)	Ko04920
10	Arginine biosynthesis	1 (7.14%)	Ko00220	25	GABAergic synapse	1 (7.14%)	Ko04727
11	Proximal tubule bicarbonate reclamation	1 (7.14%)	Ko04964	26	Epstein-Barr virus infection	1 (7.14%)	Ko05169
12	Tuberculosis	1 (7.14%)	Ko05152	27	Phagosome	1 (7.14%)	Ko04145
13	Glucagon signaling pathway	1 (7.14%)	Ko04922	28	Glutamatergic synapse	1 (7.14%)	Ko04724
14	Insulin resistance	1 (7.14%)	Ko04931	29	Pathways in cancer	1 (7.14%)	Ko05200
15	Alanine, aspartate, and glutamate metabolism	1 (7.14%)	Ko00250	30	RNA transport	1 (7.14%)	Ko03013

## Data Availability

Transcriptome raw data was published in [32], which is deposited in NCBI (https://www.ncbi.nlm.nih.gov/sra/SRP129841, SRA accession: SRP129841; Temporary Submission ID: SUB3501735) [32]; proteomics raw data was published in [33], which is available via ProteomeXchange with identifier PXD009179 (username: reviewer55751@ebi.ac.uk, password: k5zenarO) [33].

## Supplementary Materials

Supplementary Materials Table S1: statistics of pathway enrichment correlation. Figure S1: GO analyses of correlations for Quant. Figure S2: GO analyses of correlations for DEPs_NDEGs. Figure S3: GO analyses of correlations for NDEPs_NDEGs. Figure S4: COG function classification of correlations for Quant (A), DEPs_NDEGs (B), NDEPs_DEGs (C), and NDEPs_NDEGs (D). Figure S5: bile secretion (Ko04976). Figure S6: proximal tubule bicarbonate (Ko04964).
