# Supplementary material for: Gene Identification and Characterization of Correlations for DEPs_DEGs Same Trend Responding to Salinity Adaptation in Scylla paramamosain
Source: Int J Genomics. 2019 Feb 10;2019:7940405. doi: 10.1155/2019/7940405 (PMC6387702; doi:10.1155/2019/7940405)
Supplement: Supplementary Materials — Table S1: statistics of pathway enrichment correlation. Figure S1: GO analyses of correlations for Quant. Figure S2: GO analyses of correlations for DEPs_NDEGs. Figure S3: GO analyses of correlations for NDEPs_NDEGs. Figure S4: COG function classification of correlations for Quant (A), DEPs_NDEGs (B), NDEPs_DEGs (C), and NDEPs_NDEGs (D). Figure S5: bile secretion (Ko04976). Figure S6: proximal tubule bicarbonate (Ko04964). [file 7940405.f1.docx]

**Supplementary information**

**Table S1 Statistics of pathway** **enrichment correlation**

| Group Name | Type | Number of Pathway [Proteome] | Number of Pathway [Transcritome] | Number of Correlation |
| --- | --- | --- | --- | --- |
| LS-VS-CK | ALL | 279 | 164 | 152 |
|  | Significant | 21 | 8 | 3 |

**
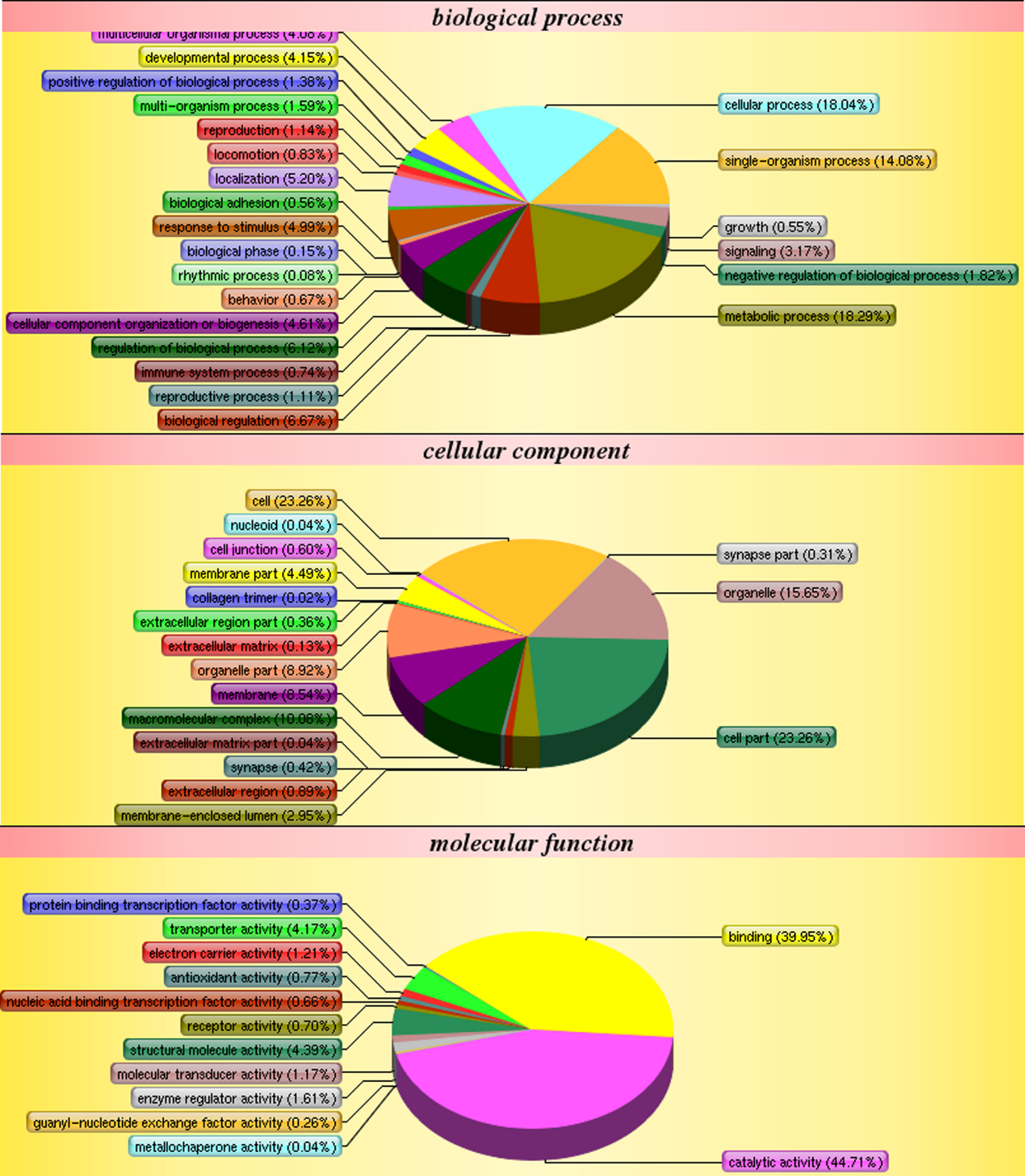
**

**Fig. S1 GO analyses of correlations for Quant**

**
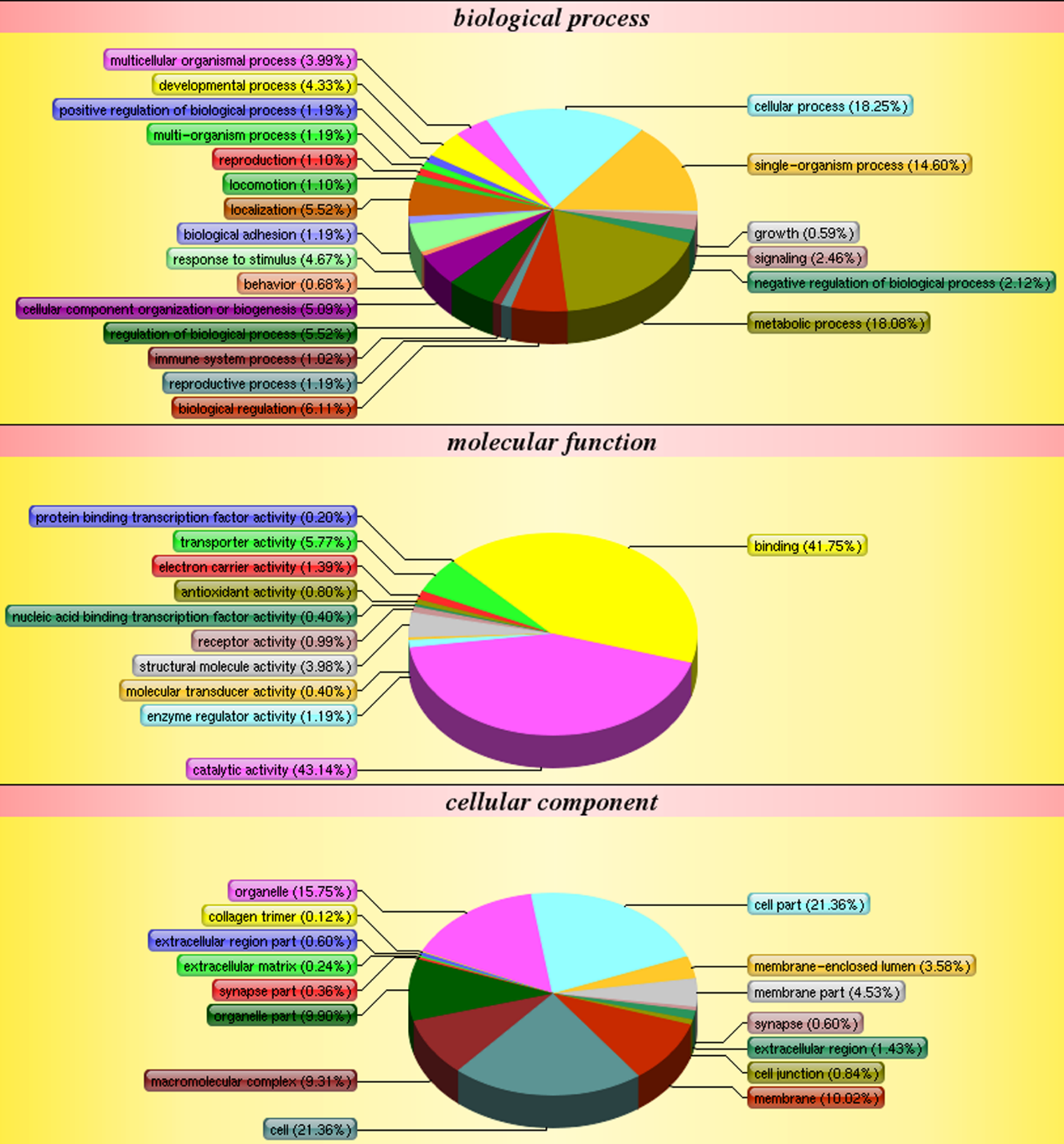
**

**Fig. S2 GO analyses of correlations for DEPs_NDEGs**

**
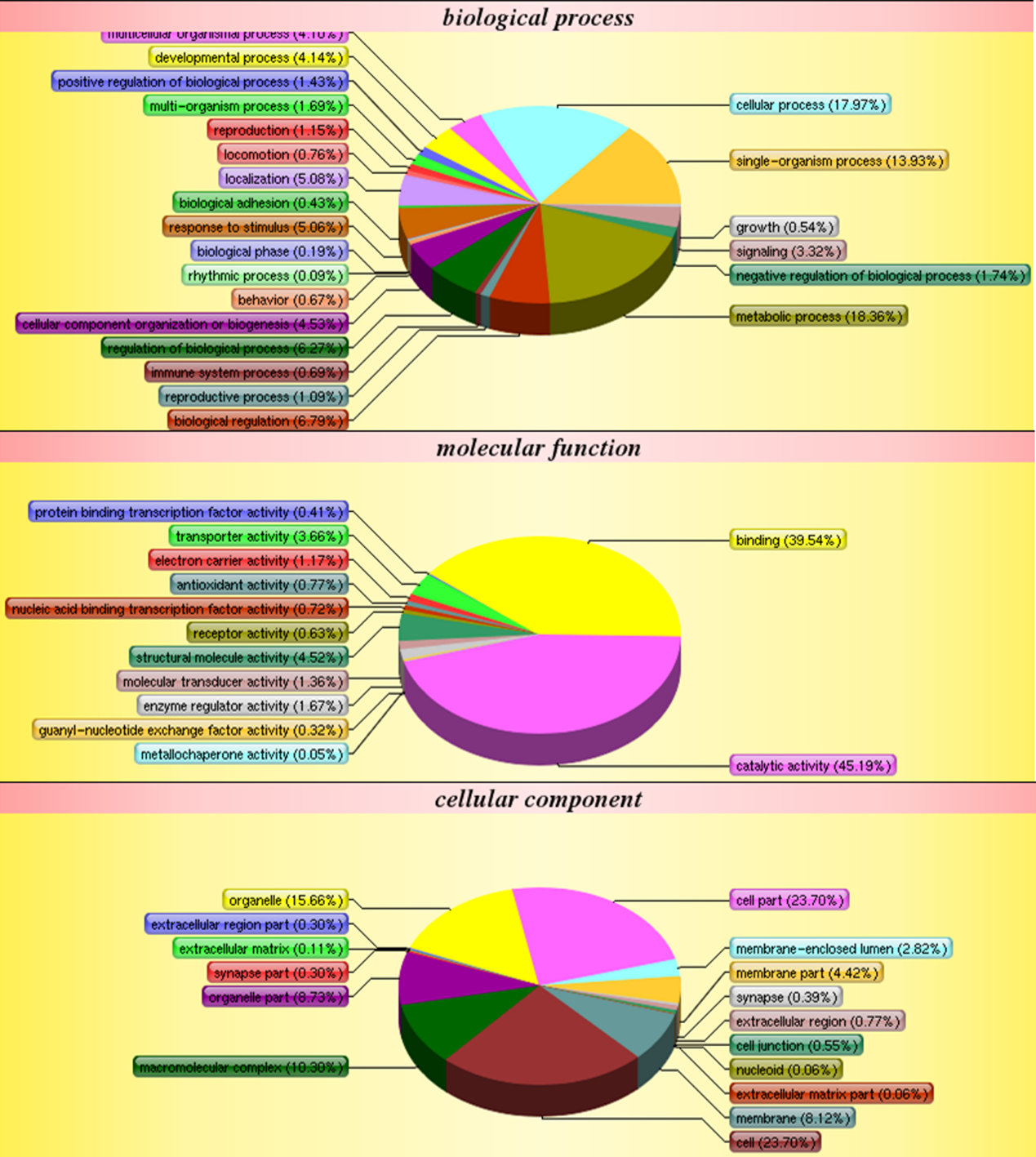
**

**Fig. S3 GO analyses of correlations for NDEPs_NDEGs**

**
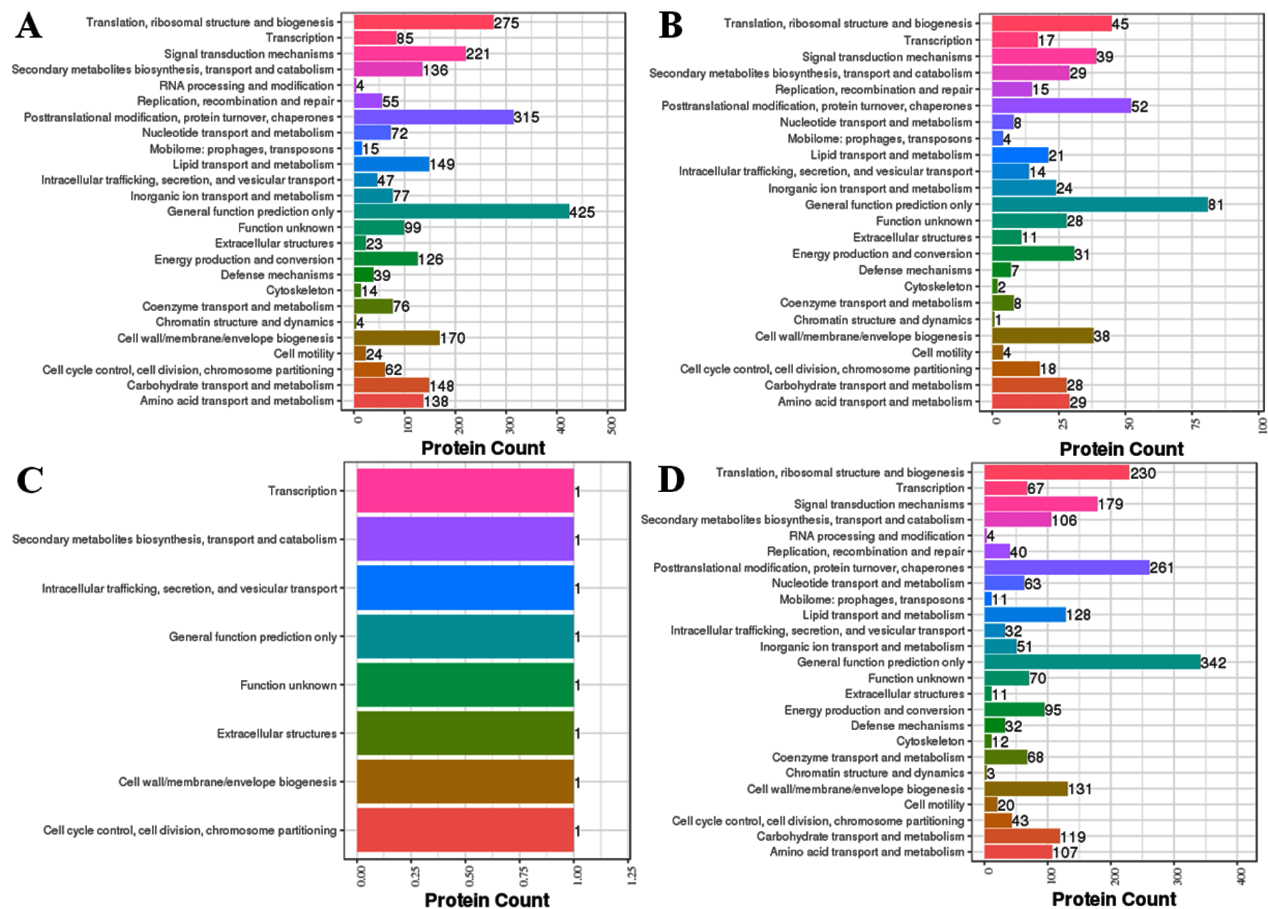
**

**Fig. S4 COG function classification of correlations for Quant (A), DEPs_NDEGs (B), NDEPs_DEGs (C), and NDEPs_NDEGs (D).**

**
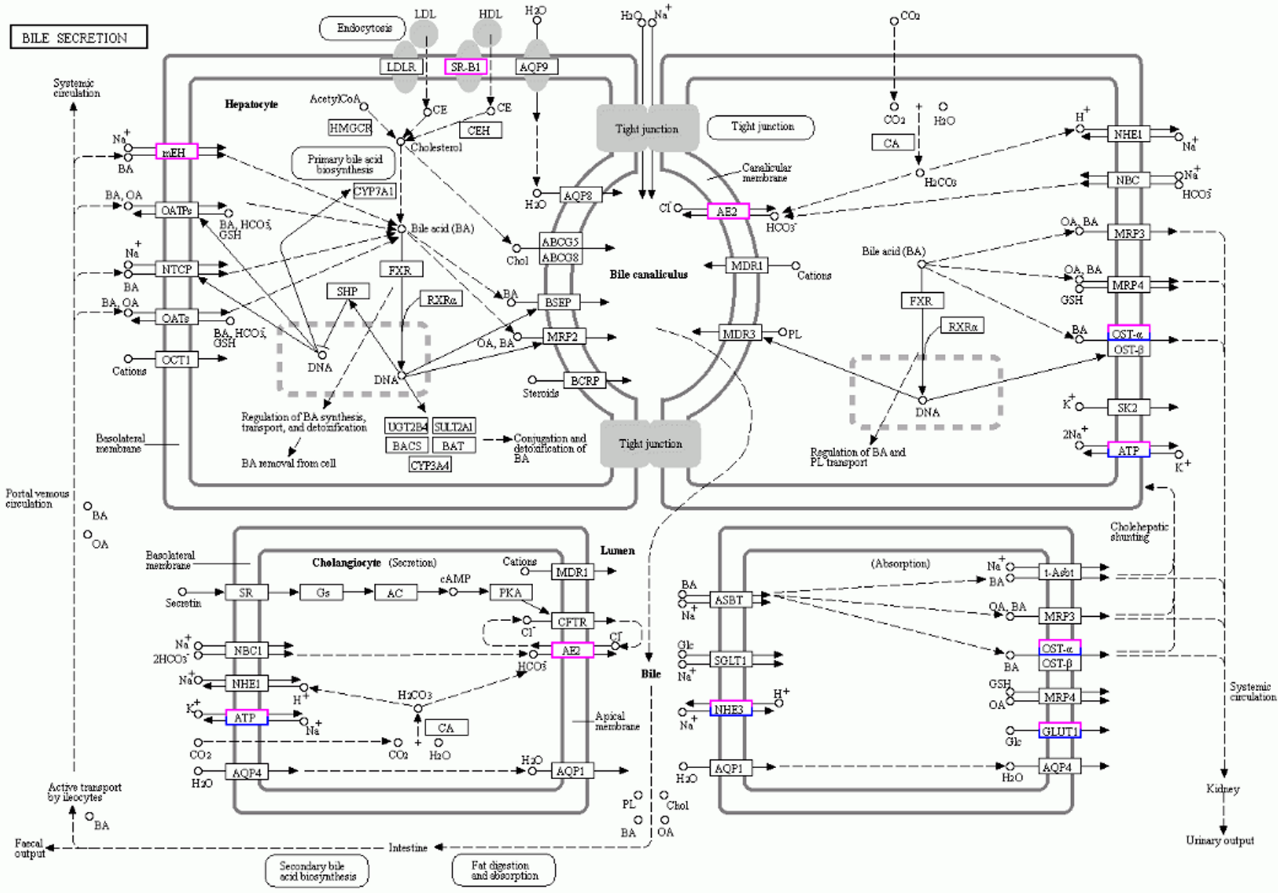
**

**Fig. S5 Bile secretion (Ko04976)**

**
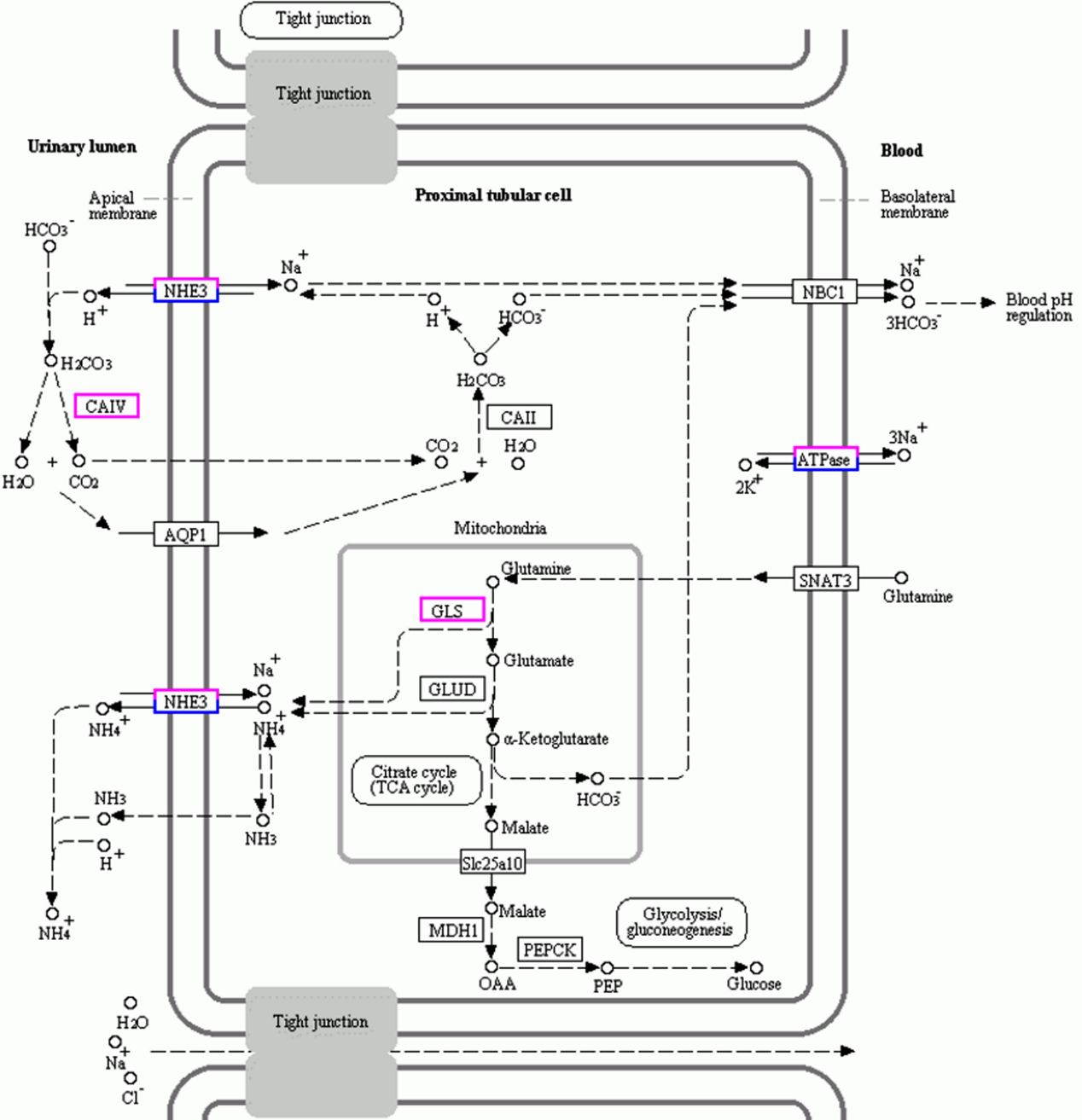
**

**Fig. S6 Proximal tubule bicarbonate (Ko04964)**
